# Supplementary material for: A Novel Antimicrobial Mechanism of Azalomycin F Acting on Lipoteichoic Acid Synthase and Cell Envelope
Source: Molecules. 2024 Feb 14;29(4):856. doi: 10.3390/molecules29040856 (PMC10893547; doi:10.3390/molecules29040856)
Supplement: Supplementary file 1 [file molecules-29-00856-s001.zip › molecules-2802498-supplementary.pdf]

Supplementary Materials

A Novel Antimicrobial Mechanism of Azalomycin F Acting on Lipoteichoic Acid Synthase and Cell Envelope

Zilong Luo [1,†], Pingyi Li [1,†], Duoduo Zhang [1,†], Jianping Zhu [2], Wan Wang [3], Wenjia Zhao [1], Peibo Li [4] and Ganjun Yuan [1,2,*]

[1] Biotechnological Engineering Center for Pharmaceutical Research and Development, Jiangxi Agricultural University, Nanchang 330045, China; zilong5716@163.com (Z.L.); 18270928169@163.com (P.L.); blossom_xin@163.com (D.Z.); 18770910458@sohu.com (W.Z.)

[2] Laboratory of Natural Medicine and Microbiological Drug, College of Bioscience and Bioengineering, Jiangxi Agricultural University, Nanchang 330045, China; zjp13698090160@163.com

[3] School of Basic Medicine, Nanchang Medical College, Nanchang 330006, China; wangwang.jeff@foxmail.com

[4] School of Life Sciences, Sun Yat-sen University, Guangzhou 510275, China; lipeibo@mail.sysu.edu.cn

* Correspondence: gyuan@jxau.edu.cn; Tel.: +86-0791-8381-3459

† These authors contributed equally to this work.

Contents

1. Experiments Details

Calculations for the quenching constants, the thermodynamic parameters and the Hill's coefficients

2. Figures

Figure S1. Basic analyses for the mass spectrometry data from the proteome of *S. aureus* treated with and without azalomycin F.

Figure S2. Schematic diagram of a KEGG pathway (as peptidoglycan biosynthesis) which significantly enriched differentially expressed proteins.

Figure S3. The expression levels of six proteins related to the LTA and cell envelope of *S. aureus* were verified using the PRM technology.

Figure S4. Enzyme activities of eLtaS (left), LtaS (middle) and LtaS-embedded liposome (right).

Figure S5. Stern-Volmer (left) and double logarithmic (right) plots of eLtaS quenched by azalomycin F respectively at 25°C (298 K) and 35°C (308 K) in five incubation systems.

Figure S6. Stern-Volmer (left) and double logarithmic (right) plots of eLtaS quenched by azalomycin F respectively at 25°C (298 K) and 35°C (308 K) in five incubation systems.

Figure S7. Synchronous fluorescence spectra of eLtaS and LtaS quenched by azalomycin F at 25°C.

Figure S8. Amino acid residues and sites of azalomycin F (AZF) binding to the LtaS.

3. Tables

Table S1. Amino acid residues of LtaS interacting with azalomycin F by ESI MS.

Table S2. The molecular docking of the guanidyl side chain of azalomycin F (AZF) and eLtaS.

Table S3. List of TOF/MS parameters.

1. Experimental Details

Calculations for the quenching constant, thermodynamic parameters, and Hill's coefficient

To explore the quenching mechanism of azalomycin F to eLtaS or LtaS, the fluorescence quenching was analyzed according to the Stern–Volmer equation as follows:

$$F_0 \ / \ F = 1 + K_{sv}[Q] = 1 + K_q\tau_0[Q] \tag{1}$$

where F_0 and F represent the maximum fluorescence intensity of eLtaS or LtaS in the absence and presence of azalomycin F, respectively; τ_0 is the average lifetime of fluorescent molecule without azalomycin F, approximately 10^{-8} s [35]; K_{sv} is the Stern–Volmer quenching constant, and K_q is the bimolecular quenching rate constant; $[Q]$ is the concentration of azalomycin F.

Based on the linear regression plots of F_0 / F versus $[Q]$ at different temperatures, the values of K_{sv} and K_q can be obtained from the corresponding slope.

To understand the binding constant and the number of binding sites of eLtaS or LtaS interacting with azalomycin F, the static quenching interaction was analyzed according to the double logarithm equation as follows [31]:

$$\lg[(F_0 - F) \ / \ F] = \lg K_a + n\lg[Q] \tag{2}$$

where F_0 and F represent the maximum fluorescence intensity of eLtaS or LtaS in the absence and presence of azalomycin F, respectively. K_a is the binding constant, n is the number of binding sites between every eLtaS or LtaS and azalomycin F molecules, and $[Q]$ is the concentration of azalomycin F.

Based on the double logarithmic plots of $\lg[(F_0 - F)/F]$ versus $\lg[Q]$ at different temperatures, the values of K_a and n can be obtained from the corresponding slope [32,33].

To elucidate the binding forces of eLtaS or LtaS interacting with azalomycin F, the thermodynamic parameters for each system were calculated from the van't Hoff equation [28,35,45]:

$$\ln(K_{a2} \ / \ K_{a1}) = \Delta H(1 \ / \ T_1 - 1 \ / \ T_2) \ / \ R \tag{3}$$

$$\Delta G = -RT \ln K_a \tag{4}$$

$$\Delta S = -(\Delta G - \Delta H) \ / \ T \tag{5}$$

where Ka is the binding constant, and T is the temperature; K_{a1} and K_{a2} are the binding constants for a system at two different temperatures T_1 and T_2; R is the gas constant, equal to 8.314 J/(mol·k); ΔH, ΔG and ΔS are the enthalpy change, Gibbs free energy and entropy change, respectively.

In biochemistry, the binding of a ligand molecule at one site of a macromolecule often influences the affinity for other ligand molecules at additional sites, and this is known as cooperative effect which can be quantitatively analyzed by Hill's coefficient (n_H). Thereby, the n_H for each incubation system was analyzed according to Hill's equation as follows [46]:

$$\lg[Y \ / \ (1 - Y)] = \lg K_a + n_H \lg[Q] \tag{6}$$

where Y is the fraction of binding saturation, namely the fraction of sites occupied by the ligand; K_a is the binding constant, and $[Q]$ is the concentration of azalomycin F; n_H is the Hill's coefficient.

Based on the linear regression plots of $\lg[Y/(1 - Y)]$ versus $\lg[Q]$ for each system at different temperature, the n_H can be obtained from the corresponding slope.

For Hill's equation, $Y/(1\text{-}Y)$ can be calculated from L and L_m according to the following formula [46]:

$$Y \ / \ (1 - Y) = L \ / \ (L_m - L) \tag{2}$$

$$L = 1 - F \ / \ F_0 \tag{3}$$

$$1/L = \mathrm{N} + 1/L_m \cdot 1 \ / \ [Q] \tag{4}$$

where F_0 and F represent the maximum fluorescence intensity of eLtaS or LtaS in the absence and presence of azalomycin F, respectively; $1/L_m$ is the slope of the linear regression plot for $1/L$ versus $1/[Q]$, and N is the intercept of the corresponding plot.

2. Figures

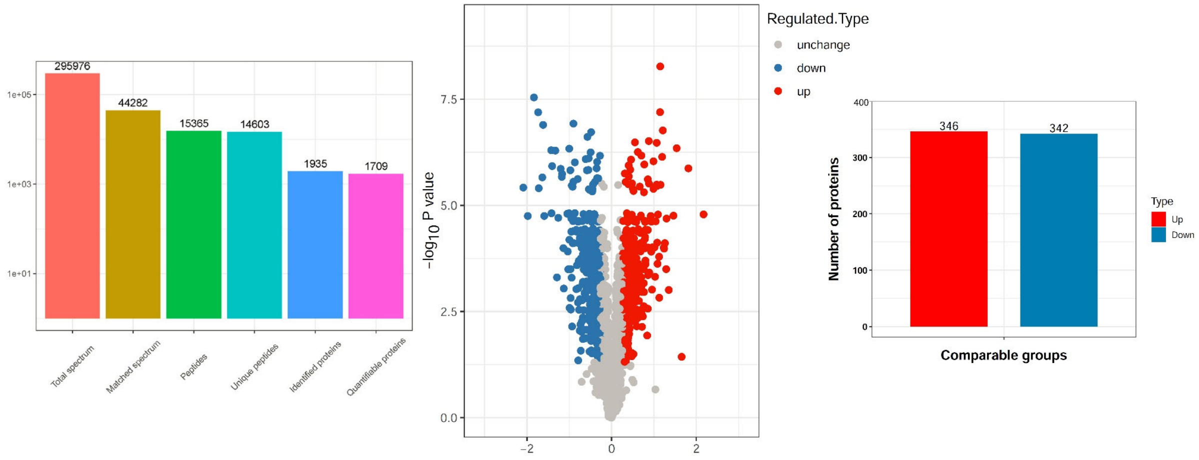

Figure S1. Basic analyses for the mass spectrometry data from the proteome of *S. aureus* treated with and without azalomycin F (n = 3). Left, statistical chart of the mass spectrometry data; Middle, volcano map for the quantification of differentially expressed proteins; Right, column chart for the distribution of differentially expressed proteins.

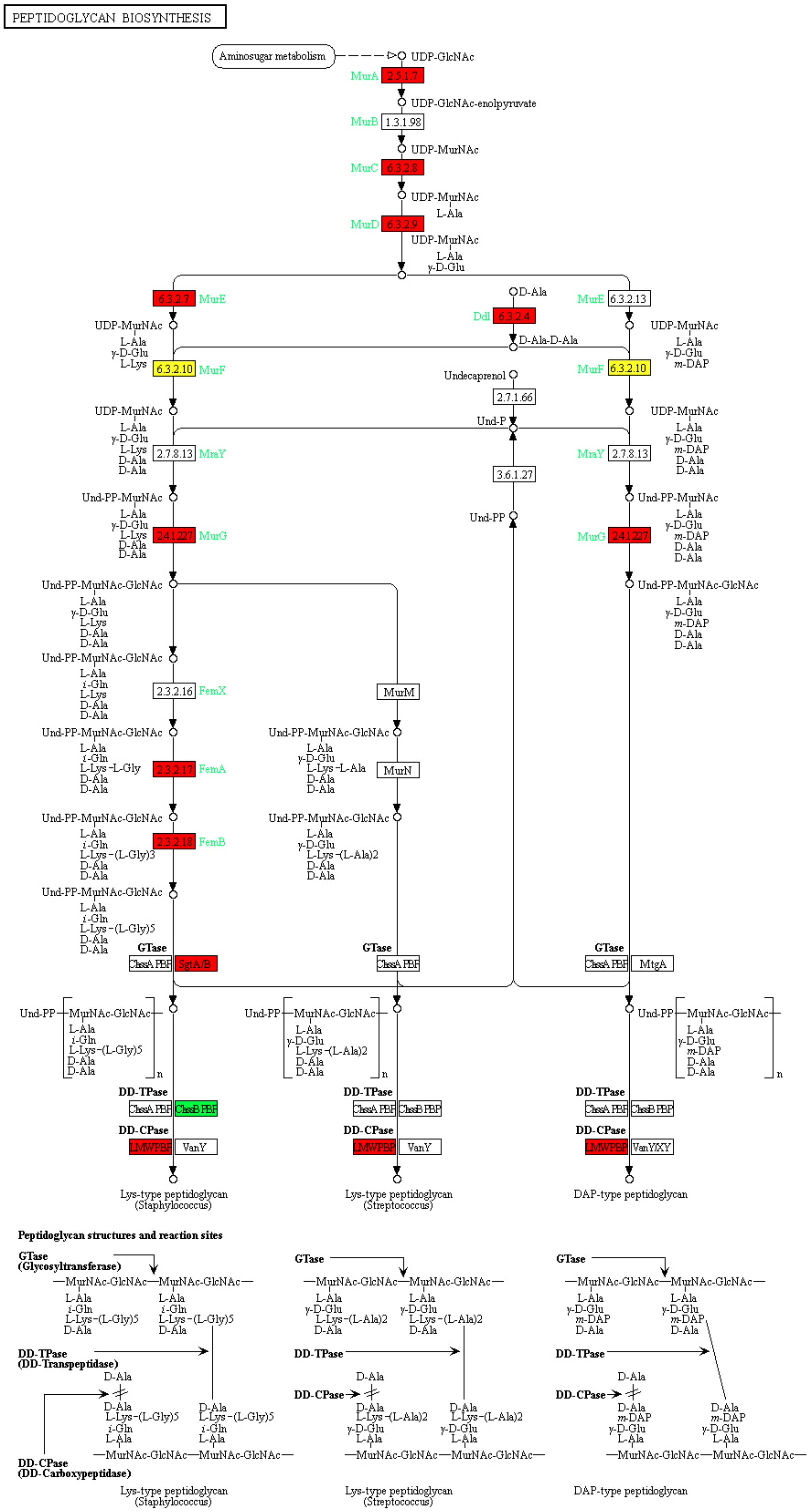

Figure S2. Schematic diagram of a KEGG pathway (as peptidoglycan biosynthesis) which significantly enriched differentially expressed proteins. Red, represents upregulated differential proteins; Green, represents downregulated differential proteins; Yellow, indicates multiple proteins presented in this node, including upregulated and downregulated differential proteins.

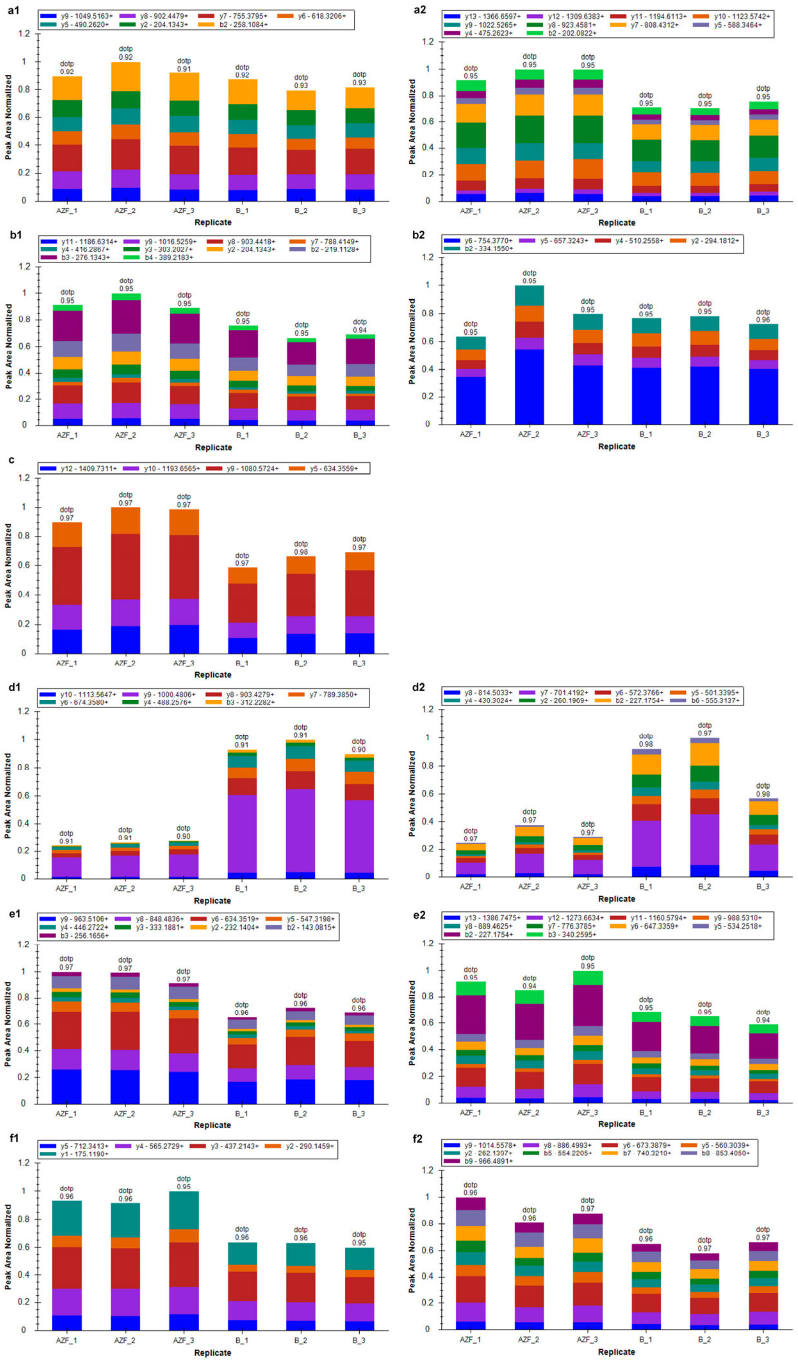

Figure S3. The expression levels of six proteins related to the LTA and cell envelope of *S. aureus* were verified using the PRM technology (n = 3). AZF_1, _2 and _3 means azalomycin F groups 1, 2 and 3; B_1, _2 and _3 means blank groups 1, 2 and 3; **a1** and **a2** were respectively peak areas of fragment ions EQFTYFPNFFHQTGQGK and SNTGDATVDGYIQTAR from protein Q2G093; **b1** and **b2** were respectively those of fragment ions AFGLIDEDQIVGK and FWPFSEFK from protein Q2FZT7; **c** was peak areas of fragment ion IIEFSELGEFIYQPVK from protein Q2G2L1; **d1** and **d2** were respectively those of fragment ions VVIPNDVSNQAR and LLEAAGLIK from protein Q2G0V0; **e1** and **e2** were respectively those of fragment ions AAIDTLSTLTGR and ILILGDVLELGENSK from protein Q2FWH4; and **f1** and **f2** were respectively those of fragment ions EAFQFDR and SAEHEVSILTAQNVLNAIDK from protein Q2FWH3.

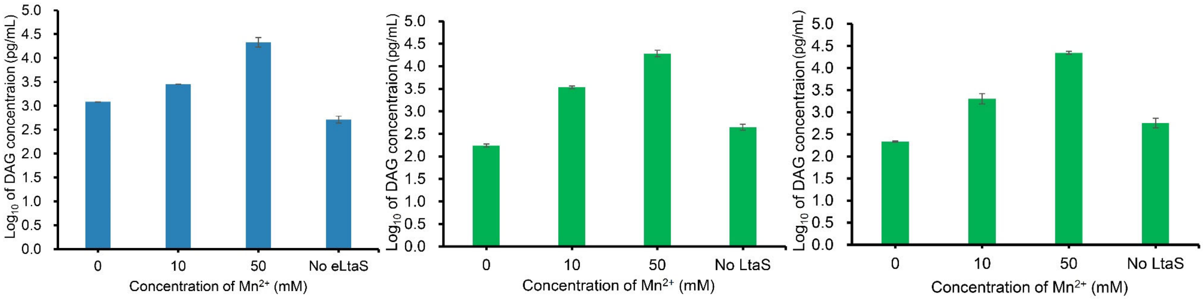

Figure S4. Enzyme activities of eLtaS (Left), LtaS (Middle) and LtaS-embedded liposome (Right) (n = 3). The concentrations of eLtaS, LtaS and LtaS-embedded liposome were respectively 2.0, 0.75 and 0.75 μM.

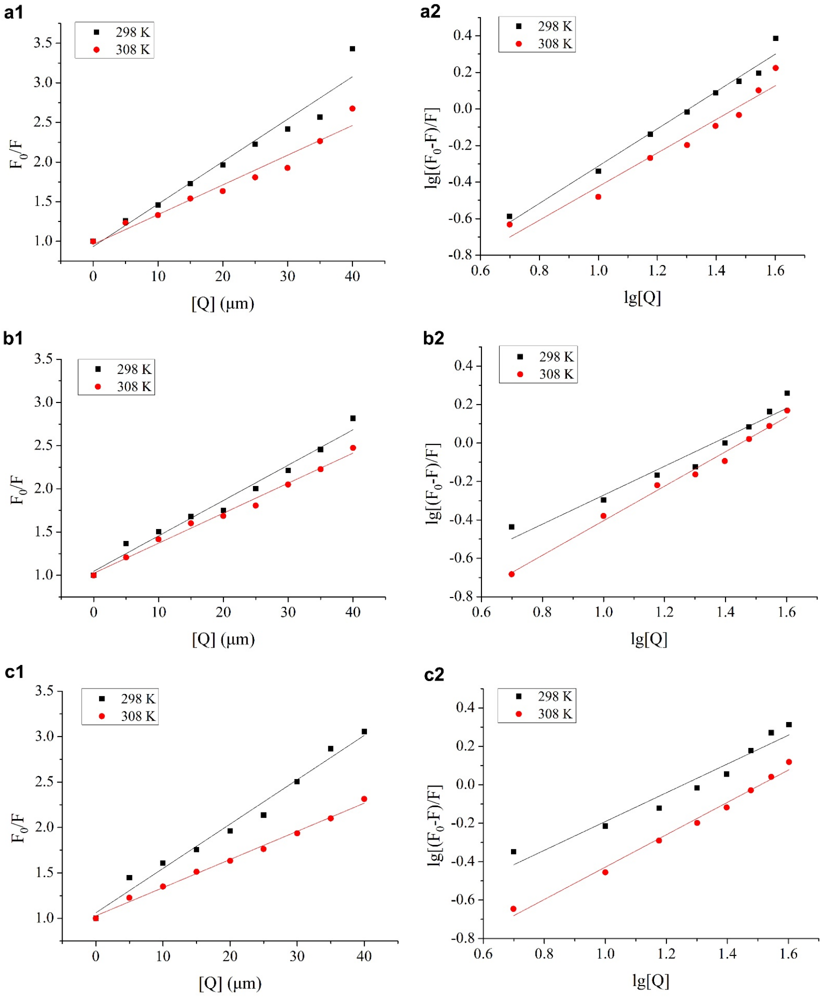

Figure S5. Stern-Volmer (left) and double logarithmic (right) plots of eLtaS quenched by azalomycin F respectively at 25°C (298 K) and 35°C (308 K) in five incubation systems. **a1** and **a2**, **b1** and **b2**, and **c1** and **c2** correspond to systems (A) eLtaS/azalomycin F, (B) eLtaS/DPPG/azalomycin F and (C) eLtaS/DPPG/MnCl2/azalomycin F, respectively.

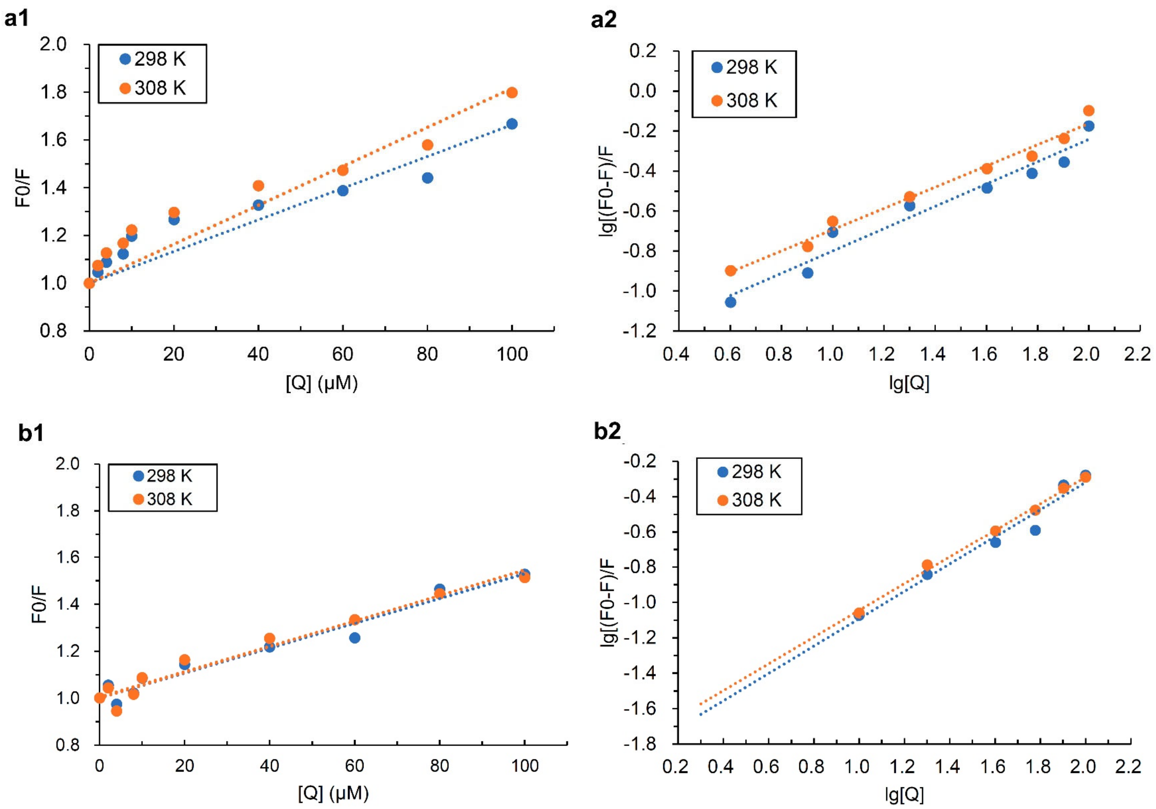

Figure S6. Stern-Volmer (left) and double logarithmic (right) plots of eLtaS quenched by azalomycin F respectively at 25°C (298 K) and 35°C (308 K) in five incubation systems. **a1** and **a2**, and **b1** and **b2** correspond to systems (D) LtaS-embedded liposome/azalomycin F, and (E) LtaS-embedded liposome/$MnCl_2$/azalomycin F, respectively.

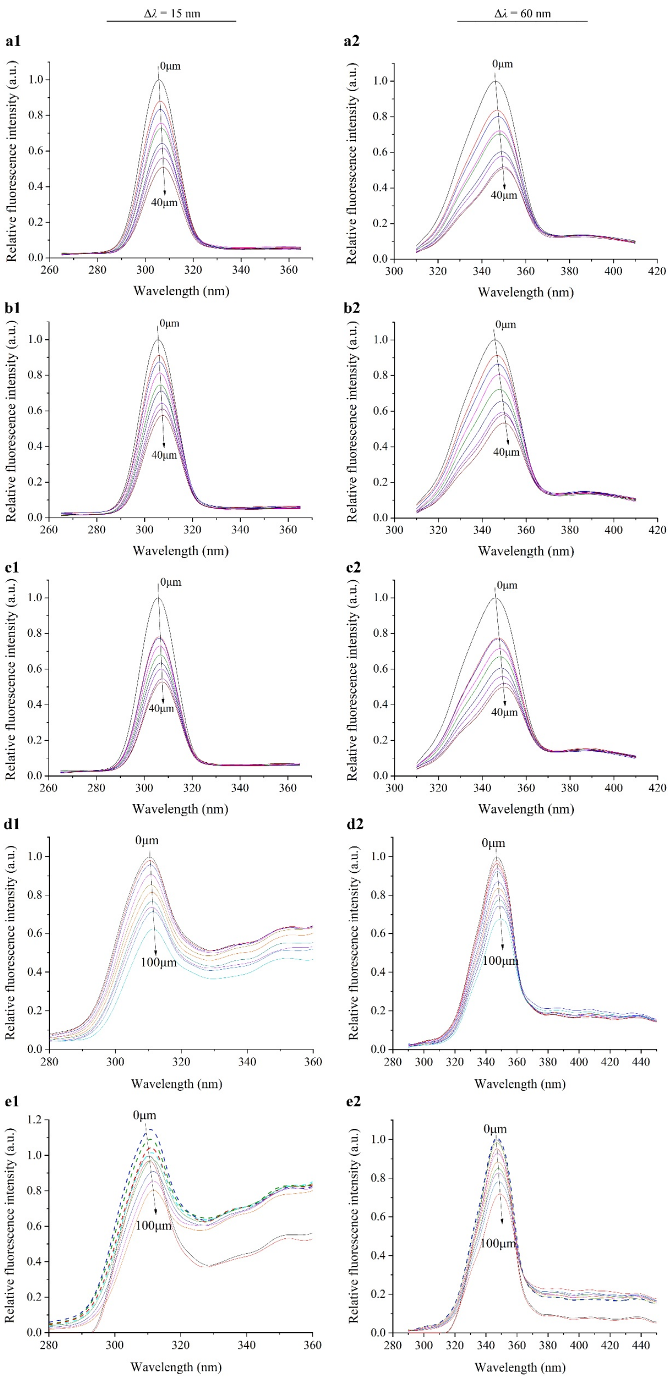

Figure S7. Synchronous fluorescence spectra of eLtaS and LtaS quenched by azalomycin F at 25°C. Systems (A), eLtaS/azalomycin F; (B), eLtaS/DPPG/azalomycin F; (C), eLtaS/DPPG/$MnCl_2$/azalomycin F; (D), LtaS-embedded liposome/azalomycin F; and (E), LtaS-embedded liposome/$MnCl_2$/azalomycin F. The tyrosine synchronous fluorescence spectra **a1** to **e1** of left column corresponded systems (A) to (E), with the wavelength difference ($\Delta\lambda$) kept as 15 nm, and while the tryptophan synchronous fluorescence spectra **a2** to **e2** of right column corresponded systems (A) to (E), with the $\Delta\lambda$ kept as 60 nm. For systems (A) to (C), the concentrations of azalomycin F were respectively 0, 5, 10, 15, 20, 25, 30, 35 and 40 μM, and those of eLtaS were 5 μM. For systems (D) and (E), the concentrations of azalomycin F were respectively 0, 2, 4, 8, 10, 20, 40, 60, 80 and 100 μM, and those of LtaS-embedded liposome were 5 μM.

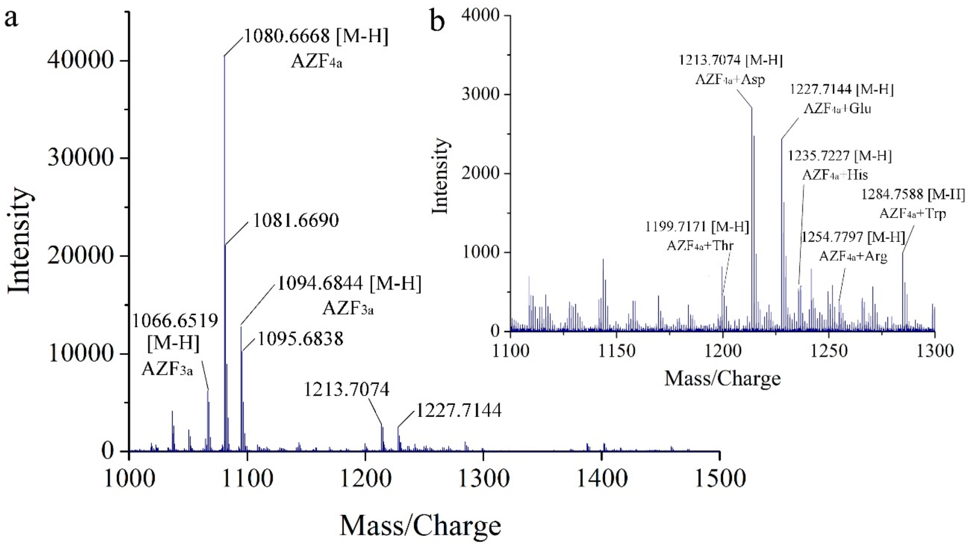

Figure S8. Amino acid residues and sites of azalomycin F (AZF) binding to the LtaS. **a**: electrospray ionization mass spectrometry analyses for the binding of azalomycin F to the amino acid residues of LtaS in negative ion mode; **b**: amplification for the mass-to-charge ratios from 1,100 to 1,300 in the mass spectra **a**.

3. Supplementary Tables

Table S1: Amino acid residues of LtaS interacting with azalomycin F by ESI MS.

Complex[a]	**[M-H]$^-$** (*m*/*z*)			**M$^+$** (*m*/*z*)		
	**Observed**	**Calculated**	**Intensity**	**Observed**	**Calculated**	**Intensity**
AZF$_{4a}$/Aspartic acid	1213.7074	1213.6958	2833	1214.7105	1214.7037	2319
AZF$_{4a}$/Glutamic acid	1227.7144	1227.7115	2433	1228.7283	1228.7193	1600
AZF$_{4a}$/Tryptophan	1284.7588	1284.7482	986	1285.7655	1285.7560	623
AZF$_{4a}$/Histidine	1235.7227	1235.7278	502	1236.7398	1236.7356	571
AZF$_{4a}$/Threonine	1199.7171	1199.7166	447	1200.7291	1200.7244	285
AZF$_{4a}$/Arginine	1254.7797	1254.7700	406	1255.7846	1255.7779	334
AZF$_{4a}$/Phenylalanine	1245.7335	1245.7373	185	1246.7497	1246.7451	207
AZF$_{4a}$/Tyrosine	1261.7425	1261.7322	114	1262.7552	1262.7400	67
DMAZF$_{4a}$/Glutamic acid	1141.7235	1141.7111	407	1142.7203	1142.7189	422
DMAZF$_{4a}$/Aspartic acid	1127.7055	1127.6954	377	1128.7103	1128.7033	333
DMAZF$_{4a}$/Threonine	1113.7175	1113.7162	157	1114.7208	1114.7240	90
DMAZF$_{4a}$/Tryptophan	1198.7495	1198.7478	149	1199.7611	1199.7556	205
DMAZF$_{4a}$/Phenylalanine	1159.7367	1159.7369	116	1160.7365	1160.7447	42
DMAZF$_{4a}$/Histidine	1149.7290	1149.7274	105	1150.7340	1150.7352	95
DMAZF$_{4a}$/Arginine	1168.7792	1168.7696	99	1169.7829	1169.7774	98
DMAZF$_{4a}$/Tyrosine	1175.7414	1175.7318	32	1176.7384	1176.7397	61

[a]: AZF$_{4a}$, azalomycin F$_{4a}$; DMAZF$_{4a}$, demalonyl azalomycin F$_{4a}$.

Table S2: The molecular docking of the guanidyl side chain of azalomycin F (AZF) and eLtaS.

Residue[a]	Action or Effect	Whole Molecule of AZF			Guanidyl Side Chain of AZF		
		AZF$_{3a}$	AZF$_{4a}$	AZF$_{5a}$	AZF$_{3a}$	AZF$_{4a}$	AZF$_{5a}$
**Lys299**	Stabling the LTA growth	---- [b]	----	----	----	----	----
Thr300	Catalytic residue binding to Mn^{2+}						
Glu255				----	----	----H	----
Asp475	Binding to Mn^{2+}			----			
His476		----	----		----		
**His416**	Binding to the substrates and protonating groups	----	----	----	----	----	----
His347				----	----H [c]	----	
Asp349				----			----
Phe353	Binding to the substrates	----	----	----	----		----
**Trp354**		----	----	----	----	----	----
Arg356				----	----H		
Leu384					----		----
Tyr477	Stabling the LTA growth		----	----			
Gly478		----	----	----			----
Ser480		----	----				
Ala300	Close to the residues of eLtaS active center	----		----		----	
Ser256		----			----		----
Leu413				----	----	----	
Tyr417					----	----	----

[a]: Amino acid residues of eltaS active center. Those marked bold indicated that there were interactions with the guanidyl side chain of azalomycin F for all dockings, and those marked brown were not but close to the residues of eLtaS active center. [b]: ----, indicated that there were hydrophobic interactions between the residues and the guanidyl side chain. [c]: ----H, indicated that there were hydrogen bond interactions between the residues and the guanidyl side chain, and while just one can form for those docking having two hydrogen bond interactions.

Table S3. List of TOF/MS parameters [a].

**Parameters**	**Negative Mode**
ISVF	-4500 V
TEM	550 °C
Gas 1	50 psi
Gas 2	50 psi
Curtain gas	30 psi
DP MS	-80 V
CE in MS	-10 eV
CE in MS/MS	-30 eV± 15 eV
Nebulizer and auxiliary gas	Nitrogen
Scan range	50-1600 da

[a]: Ionspray voltage floating (ISVF), The turbo spray temperature (TEM), Nebulizer gas (Gas 1), Heater gas (Gas 2), Curtain gas Declustering potential (DP), Collision energy in MS (CE in MS) and Collision energy in MS/MS (CE in MS/MS), Nebulizer and auxiliary gas, and scan range for negative ionization mode.
